# Case Report: Concurrent Babesiosis and GCA/PMR

**DOI:** 10.1002/iid3.70182

**Published:** 2025-03-19

**Authors:** Xiaolin Wang, Haohua Huang, Lichang Chen, Simin Guo, Qi‐Ming Gong

**Affiliations:** ^1^ Department of Infectious Diseases, Ruijin Hospital Shanghai Jiao Tong University School of Medicine Shanghai China

**Keywords:** babesiosis, case report, giant cell arteritis, polymyalgia rheumatica

## Abstract

**Background:**

Babesiosis is a tick‐transmitted illness caused by intraerythrocytic protozoa of the genus babesia. The severity of babesiosis ranges from asymptomatic infection to fatal disease. Giant cell arteritis (GCA) and polymyalgia rheumatica (PMR) are common interrelated inflammatory disorders that almost occur in people aged over 50 years. This report presents the first case of concurrent babesiosis and GCA/PMR in an old person.

**Case Presentation:**

A 63‐year‐old man was admitted to the hospital with a 1‐month history of fevers, accompanied by headache, muscle pain and fatigue. Laboratory tests revealed hemolytic anemia, with elevated C‐reactive protein, serum IL‐6 and erythrocyte sedimentation rate. FDG positron‐emission tomography‐computed tomography (PET‐CT) scan exhibited increased uptake in aortic wall, multiple medium‐to‐large arteries and soft tissues. A blood smear revealed Babesia microti intracellular ring forms. Babesia microti infection was further confirmed by polymerase chain reaction (PCR) test. This patient was diagnosed as concurrent babesiosis and GCA/PMR. He was treated with glucocorticoid and antimicrobial therapy.

**Conclusions:**

Concurrent babesiosis and GCA/PMR is rare. Further studies are needed to understand the mechanism of interaction between babesiosis and human immune system.

## Graphical abstract

Babesiosis is a tick‐transmitted infectious disease caused by parasites of the genus *Babesia*. Giant cell arteritis (GCA) and polymyalgia rheumatica (PMR) are common interrelated inflammatory disorders. This report presents the first case of concurrent babesiosis and GCA/PMR.

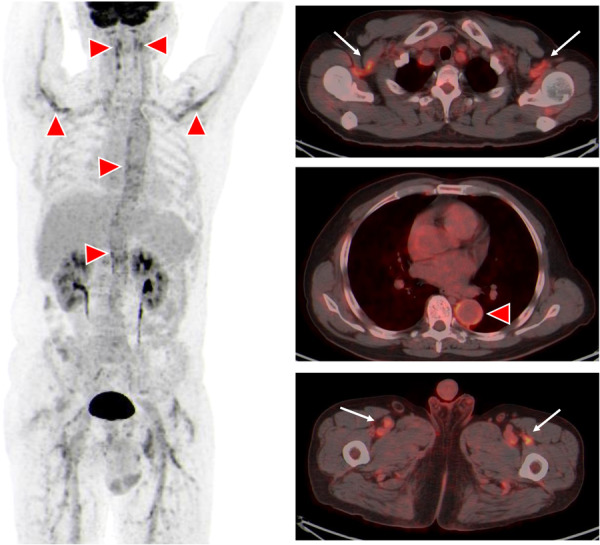

## 1 Introduction

Giant cell arteritis (GCA) and polymyalgia rheumatica (PMR) are related inflammatory disorders that predominantly impact individuals over the age of 50 and often coexist [1, 2]. GCA usually manifests as unilateral or bilateral headaches, myalgias, fatigue, fever, and weight loss, while PMR often presents acutely with pain in both upper extremities [1, 2]. Elevations of inflammatory markers, such as erythrocyte sedimentation rate (ESR) and C‐reactive protein (CRP) are important features of both GCA and PMR [1, 2]. GCA and PMR are identified through clinical manifestations, elevated inflammatory markers, and imaging studies. Positron‐emission tomography‐computed tomography (PET/CT) is a potent imaging modality in GCA and PMR that is consistent with clinical diagnosis [3].

Babesiosis is a tick‐transmitted infectious disease caused by parasites of the genus Babesia. It is primarily spread through tick bites and only occasionally via blood transfusion or organ donation. In China, the most prevalent species was found to be *Babesia microti* [4]. In general, babesia infections course with varying degrees of severity, varies from asymptomatic to severe and potentially life‐threatening. The severity of human babesiosis is often related to the host's age, immune status, and health conditions [5, 6]. Clinically manifest *Babesia* infection can include hemolytic anemia and nonspecific symptoms resembling influenza (e.g., fever, chills, myalgia, weakness, and fatigue) [7]. The coincidental GCA and PMR with babesiosis has not previously been described. Here we present a case of a patient with concurrent babesiosis and GCA/PMR.

## 2 Case Description

**Figure 1:**
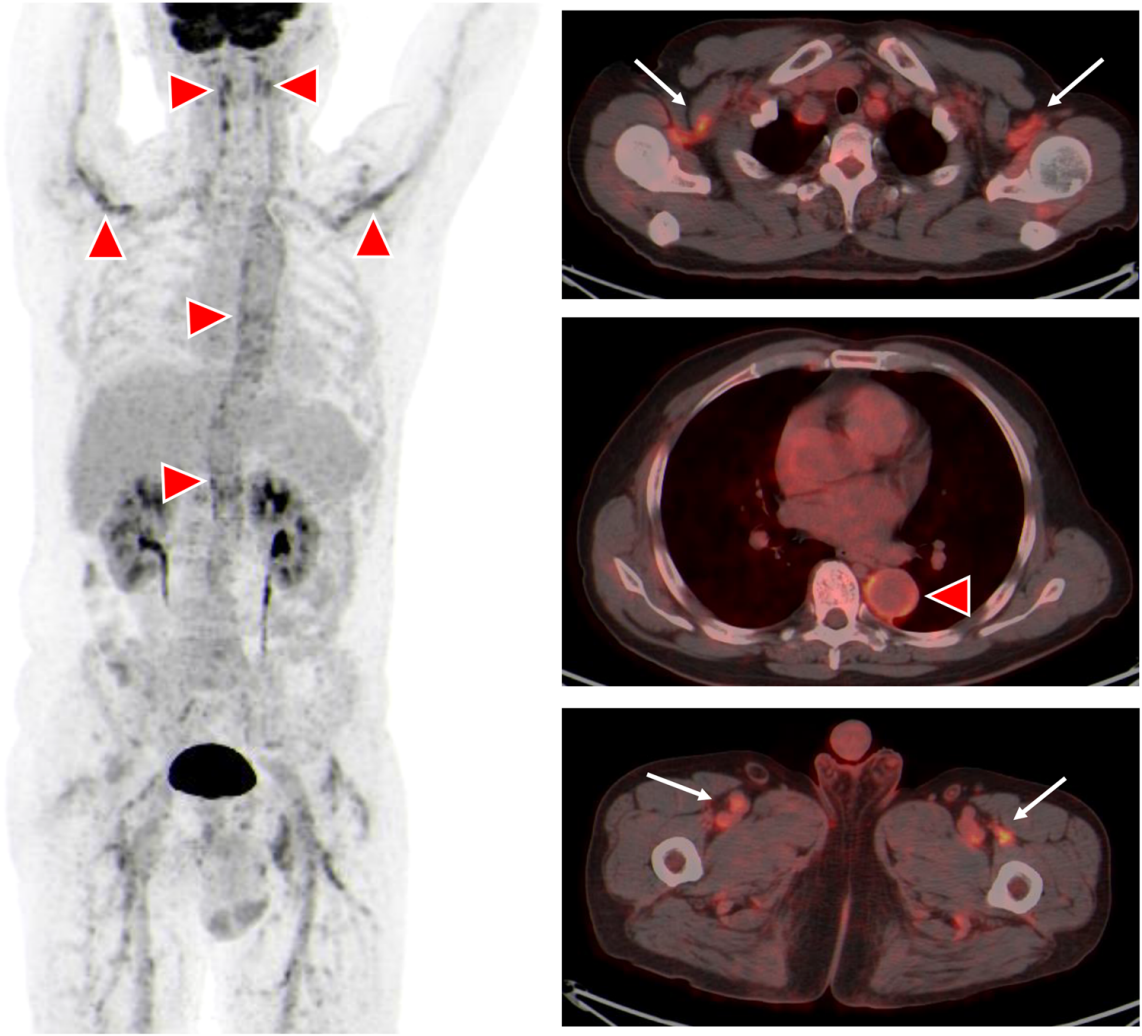
18F fluorodeoxyglucose (FDG) positron‐emission tomography‐computed tomography (PET‐CT) scan indicated giant cell arteritis and polymyalgia rheumatica. High‐level FDG uptake was shown in aortic wall, multiple medium‐to‐large arteries (arrowheads) and in soft tissues around the shoulders and hips (arrows).

**Figure 2:**
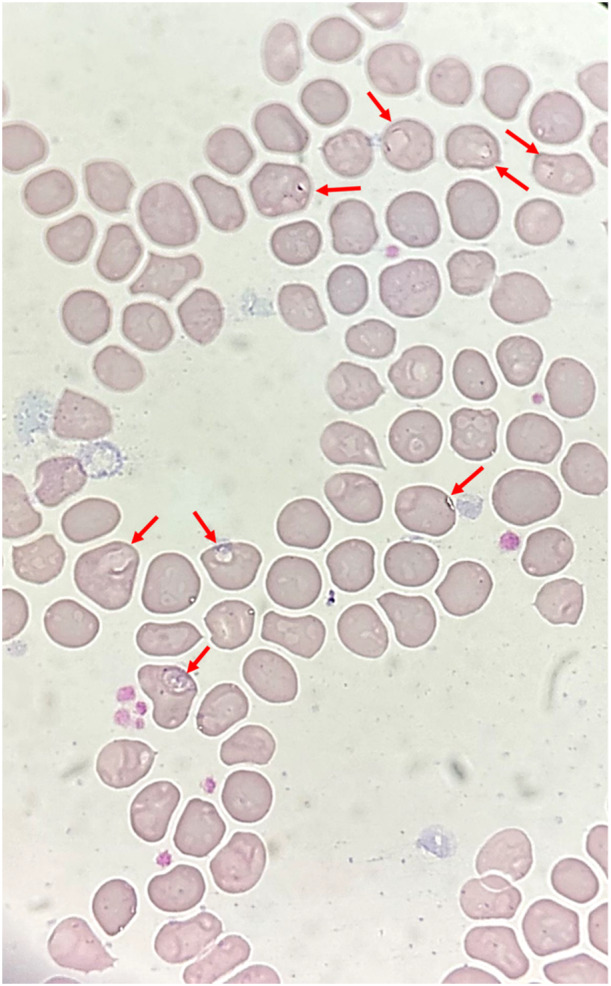
Microscopic examination of a blood smear showed ring forms of *Babesia microti*.

A 63‐year‐old Han Chinese man who often goes fishing was admitted to hospital in Shanghai, China with a 1‐month history of fevers (peak temperature, 38.3°C), accompanied by bitemporal headache, proximal myalgia of the shoulder and pelvic girdles and fatigue. He did not have symptoms of scalp tenderness, jaw claudication, visual changes and morning stiffness. Laboratory tests revealed a hemolytic anemia, with a hemoglobin level of 8.6 g per deciliter. The concentrations of serum CRP (106.0 mg/L, reference value 0–10 mg/L) and interleukin (IL)‐6 (85.7 pg/mL, reference value 0–5.4 pg/mL) were markedly increased. The liver function tests showed increased level of alkaline phosphatase (164 IU/L, reference value 45–125 IU/L) and γ‐glutamyl transferase 94 IU/L (reference value 10–60 IU/L) and other liver functions tests were normal. The level of alanine aminotransferase was 36 IU/L (reference value 9–50 IU/L), the level of aspartate aminotransferase was 24 IU/L (reference value 15–40 IU/L), and the total bilirubin level was 8.9 μmol/L (reference value 4.7–24 μmol/L). Besides, the platelets level (554 × 10^9^/L, reference value 125–350 × 10^9^/L), ESR (106 mm/h, reference value 0–15 mm/h), IgG (20.42 g/L, reference value 8.6–17.4 g/L), complement 3 (2.1 g/L, reference value 0.74–1.4 g/L), and complement 4 (0.71, reference value 0.1–0.4 g/L) were elevated. Serologic testing showed an antinuclear antibody titer of 1:160. PET‐CT scan showed that the metabolism of the aortic wall, bilateral subclavian artery, axillary artery, internal thoracic artery, common carotid artery, internal carotid artery, external carotid artery, vertebral artery, maxillary artery, superficial temporal artery, common iliac artery, internal iliac artery, external iliac artery and femoral artery was diffusely increased with an SUVmax of 7.2. The metabolism of soft tissues near shoulder joints, hip joints, trochanters and ischial tuberosities on both sides was increased with an SUVmax of 5.9. The metabolism of several interspinous ligaments in the spine was increased with an SUVmax of 4.9. The PET‐CT findings indicated giant cell arteritis and polymyalgia rheumatica. Figure 1 demonstrated the significant high‐level FDG uptake by aortic wall, multiple medium‐to‐large arteries (Figure 1, arrowheads). Increased radiolabel uptake was also shown in soft tissues around the shoulders and hips (Figure 1, arrows). Microscopic examination of a blood smear detected ring forms of Babesia microti (Figure 2). Babesia microti infection was further confirmed by polymerase chain reaction (PCR) test and by sequencing from mice inoculated with the patient's blood. This patient was diagnosed as concurrent babesiosis and GCA/PMR. He was treated with methylprednisolone (80 mg/d for 7days and 40 mg/d for another 7 days). His temperature returned to normal the second day after methylprednisolone administration. After 2 weeks, his hemoglobin level increased to 10.4 g per deciliter, the concentrations of serum CRP returned to normal level (3.5 mg/L). Besides, ESR decreased to 46 mm/h. His symptoms such as headache, muscle pain and fatigue were significantly alleviated. This patient was further treated with atovaquone and azithromycin combination therapy for 14 days. The dose of methylprednisolone was decreased to 20 mg/d and lasted for 4 weeks. Nevertheless, 1 month later, this patient experienced relapse of babesiosis despite initial clinical improvement after the first course of anti‐babesial treatment. Thus, a repeated course of anti‐babesial therapy with atovaquone and azithromycin was administrated. The dose of methylprednisolone was further decreased to 8 mg/d and lasted for 4 weeks. Whereas, 1 month after cessation of therapy, microscopic examination of blood smear detected Babesia microti again. This patient received the third course of anti‐babesial therapy with artemether and atovaquone for 14 days. The therapy of methylprednisolone was stopped. Eventually, Babesia microti was not detected in the patient's blood by PCR test again.

## 3 Discussion

*Babesia* species are intra‐erythrocytic parasites. Once *Babesia* parasites are introduced into the human host following a tick bite, the sporozoites enter erythrocytes and undergo asexual replication, inducing erythrocyte membrane disruption and ensuing hemolysis [8]. *Babesia* export numerous proteins to modify antigens on the surface of infected erythrocytes, leading to immune evasion, microvascular obstruction and facilitating cytoadherence of infected erythrocytes to vascular endothelium [9].

Following exposure to *Babesia*, the host mounts immune response as a defense, conferring protective effects but may also induce autoimmune disorders. Several studies have indicated the association between babesiosis and human auto‐immune diseases. For example, Rabah et al. [10] reported a severe case of ANCA/ANA‐positive babesiosis in an asplenic individual. Narurkar et al reported a case of babesiosis‐associated immune thrombocytopenia [11]. Besides, babesiosis has also been documented to induce warm autoimmune hemolytic anemia [12] and trigger or reactivate Evans syndrome [13, 14]. However, a case of concurrent babesiosis and GCA/PMR has not been reported in the literature.

GCA and PMR often overlap as inflammatory rheumatic diseases in the elderly [2]. The pathophysiology of GCA is featured by transmural inflammation of medium‐large‐sized arteries and chronic vascular wall damage and healing [2]. The inciting factor for the inflammatory cascade in GCA remains unknown. During the initiation phase of GCA, dendritic cells and macrophages in the adventitia become abnormally activated through pathogen‐associated molecular patterns (PAMPs) or microorganism‐associated molecular patterns (MAMPs), results in the generation of inflammatory mediators like IL‐1 and IL‐6, along with the stimulation of T lymphocyte activity [1]. Then, the intricate cyclical interactions and the intensification of inflammatory processes subsequently leads to arterial remodeling and vascular occlusion in GCA [1]. Additional research is required to comprehensively elucidate the pathogenesis of this infection and its interplay with the host immune response.

Glucocorticoid treatment is currently the preferred first‐line therapy for both GCA and PMR [1, 2]; however, glucocorticoids cause immunosuppression which may influence the outcomes of antimicrobial treatment and may lead to impaired resolution of babesiosis. Data from case reports have suggested that babesiosis may persist or relapse in patients with pre‐existing immunosuppressive conditions such as B cell lymphoma, HIV infection or solid tumor [6]. In this current case, the patient underwent glucocorticoid therapy for GCA and PMR and showed significant improvement; however, a relapsing course of babesiosis was observed. Since glucocorticoids suppress immune response [15], we speculated that the glucocorticoid therapy may compromise the efficacy of anti‐babesial therapy.

*B. microti* is found throughout the world. In China, Heilongjiang province and Guangxi province are the most infected areas [16]. Babesiosis is not endemic in Shanghai, China. Thus, in this case babesiosis should not be considered as a differential diagnosis of GCA. Although babesiosis may induce auto‐immune phenomenon, it is not reasonable to attribute the cause of GCA/PMR to babesiosis in this case. It is possible that babesiosis infection mimics GCA/PMR, since all features of this case are typical for a babesiosis infection. In this case, PET‐CT was used to diagnose GCA. Although PET‐CT is a useful noninvasive tool in the diagnosis of GCA, a temporal artery biopsy can provide more information to confirm GCA and it may help to the differential diagnosis of “primitive GCA” versus “*Babesia microti* triggered GCA.”

In this case report, we presented the first case of concurrent babesiosis and GCA and PMR. This patient received glucocorticoid therapy and three courses of anti‐babesial. More studies are needed to understand the mechanism of interaction between babesiosis and human immune system.

## Author Contributions

Xiaolin Wang and Haohua Huang wrote the manuscript. Lichang Chen and Simin Guo contributed in interpreting the patient data. Qi‐Ming Gong supervised the whole work and edited the manuscript.

## Consent

Consent for publication was obtained from the patient.

## Conflicts of Interest

The authors declare no conflicts of interest.

## References

[1] C. Dejaco , E. Brouwer , J. C. Mason , F. Buttgereit , E. L. Matteson , and B. Dasgupta , “Giant Cell Arteritis and Polymyalgia Rheumatica: Current Challenges and Opportunities,” Nature Reviews Rheumatology 13 (2017): 578–592. 28905861 10.1038/nrrheum.2017.142

[2] F. Buttgereit , E. L. Matteson , and C. Dejaco , “Polymyalgia Rheumatica and Giant Cell Arteritis,” Journal of the American Medical Association 324 (2020): 993–994. 32897333 10.1001/jama.2020.10155

[3] A. Emamifar , T. Ellingsen , S. Hess , et al., “The Utility of 18F‐FDG PET/CT in Patients With Clinical Suspicion of Polymyalgia Rheumatica and Giant Cell Arteritis: A Prospective, Observational, and Cross‐Sectional Study,” ACR Open Rheumatology 2 (2020): 478–490. 33439554 10.1002/acr2.11163 PMC7437127

[4] J. Wang , S. Zhang , J. Yang , et al., “Babesia Divergens In Human in Gansu Province, China,” Emerging Microbes & Infections 8 (2019): 959–961. 31244397 10.1080/22221751.2019.1635431 PMC6598508

[5] E. M. Bloch , S. Kumar , and P. J. Krause , “Persistence of *Babesia microti* Infection in Humans,” Pathogens 8 (2019): 102. 31319461 10.3390/pathogens8030102 PMC6789900

[6] P. J. Krause , B. E. Gewurz , D. Hill , et al., “Persistent and Relapsing Babesiosis in Immunocompromised Patients,” Clinical Infectious Diseases 46 (2008): 370–376. 18181735 10.1086/525852

[7] L. Schnittger , A. E. Rodriguez , M. Florin‐Christensen , and D. A. Morrison , “ *Babesia*: A World Emerging,” Infection, Genetics and Evolution 12 (2012): 1788–1809. 22871652 10.1016/j.meegid.2012.07.004

[8] R. M. O'Connor and D. R. Allred , “Selection of *Babesia bovis*‐Infected Erythrocytes for Adhesion to Endothelial Cells Coselects for Altered Variant Erythrocyte Surface Antigen Isoforms,” Journal of Immunology 164 (2000): 2037–2045. 10.4049/jimmunol.164.4.2037 10657656

[9] H. Hakimi , J. Yamagishi , S. Kawazu , and M. Asada , “Advances in Understanding Red Blood Cell Modifications by *Babesia* ,” PLoS Pathogens 18 (2022): e1010770. 36107982 10.1371/journal.ppat.1010770 PMC9477259

[10] H. Rabah , D. Chukkalore , E. El‐Charabaty , and N. Mobarakai , “Babesiosis and the Human Immune System,” IDCases 27 (2022): e01368. 34993053 10.1016/j.idcr.2021.e01368 PMC8713127

[11] R. Narurkar , A. Mamorska‐Dyga , A. Agarwal , J. C. Nelson , and D. Liu , “Babesiosis‐Associated Immune Thrombocytopenia,” Stem Cell Investigation 4 (2017): 1. 28217703 10.21037/sci.2017.01.02 PMC5313279

[12] A. E. Woolley , M. W. Montgomery , W. J. Savage , et al., “Post‐Babesiosis Warm Autoimmune Hemolytic Anemia,” New England Journal of Medicine 376 (2017): 939–946. 28273010 10.1056/NEJMoa1612165

[13] J. J. Shatzel , K. Donohoe , N. Q. Chu , et al., “Profound Autoimmune Hemolysis and Evans Syndrome In Two Asplenic Patients With Babesiosis,” Transfusion 55 (2015): 661–665. 25354478 10.1111/trf.12901

[14] S. A. Elder , J. J. O'Brien , Z. N. Singh , et al., “Babesiosis Masquerading as Evans Syndrome,” American Journal of Medicine 132 (2019): e616–e617. 30904506 10.1016/j.amjmed.2019.02.041

[15] D. Franchimont , J. Galon , M. Gadina , et al., “Inhibition of Th1 Immune Response by Glucocorticoids: Dexamethasone Selectively Inhibits IL‐12‐Induced Stat4 Phosphorylation In T Lymphocytes,” Journal of Immunology 164 (2000): 1768–1774. 10.4049/jimmunol.164.4.1768 10657623

[16] Z. Chen , H. Li , X. Gao , et al., “Human Babesiosis in China: A Systematic Review,” Parasitology Research 118 (2019): 1103–1112. 30770979 10.1007/s00436-019-06250-9

